# Minisyncoccus archaeiphilus gen. nov., sp. nov., a mesophilic, obligate parasitic bacterium and proposal of Minisyncoccaceae fam. nov., Minisyncoccales ord. nov., Minisyncoccia class. nov. and Minisyncoccota phyl. nov. formerly referred to as Candidatus Patescibacteria or candidate phyla radiation

**DOI:** 10.1099/ijsem.0.006668

**Published:** 2025-02-07

**Authors:** Meri Nakajima, Ryosuke Nakai, Yuga Hirakata, Kengo Kubota, Hisashi Satoh, Masaru K. Nobu, Takashi Narihiro, Kyohei Kuroda

**Affiliations:** 1 Bioproduction Research Institute, National Institute of Advanced Industrial Science and Technology (AIST), 2-17-2-1 Tsukisamu-Higashi, Toyohira-ku, Sapporo, Hokkaido 062-8517, Japan; 2 Division of Environmental Engineering, Faculty of Engineering, Hokkaido University, North-13, West-8, Sapporo, Hokkaido 060-8628, Japan; 3 Bioproduction Research Institute, National Institute of Advanced Industrial Science and Technology (AIST), Central 6, Higashi 1-1-1, Tsukuba, Ibaraki 305-8566, Japan; 4 Department of Civil and Environmental Engineering, Graduate School of Engineering, Tohoku University, 6-6-06 Aza-Aoba, Aramaki, Aoba-ku, Sendai, Miyagi 980-8579, Japan; 5 Department of Frontier Sciences for Advanced Environment, Graduate School of Environmental Studies, Tohoku University, 6-6-06 Aza-Aoba, Aramaki, Aoba-ku, Sendai, Miyagi 980-8579, Japan; 6 Institute for Extra-cutting-edge Science and Technology Avant-garde Research (X-star), Japan Agency for Marine-Earth Science and Technology (JAMSTEC), 2-15 Natsushima-cho, Yokosuka, Kanagawa 237-0061, Japan

**Keywords:** candidate phyla radiation (CPR), co-culture, methanogen, parasitism, Patescibacteria

## Abstract

In the domain *Bacteria*, one of the largest, most diverse and environmentally ubiquitous phylogenetic groups, *Candidatus* Patescibacteria (also known as candidate phyla radiation/CPR), remains poorly characterized, leaving a major knowledge gap in microbial ecology. We recently discovered a novel cross-domain symbiosis between *Ca*. Patescibacteria and *Archaea* in highly purified enrichment cultures and proposed *Candidatus* taxa for the characterized species, including *Ca*. Minisyncoccus archaeophilus and the corresponding family *Ca*. Minisyncoccaceae. In this study, we report the isolation of this bacterium, designated strain PMX.108^T^, in a two-strain co-culture with a host archaeon, *Methanospirillum hungatei* strain DSM 864^T^ (JF-1^T^), and hereby describe it as the first representative species of *Ca*. Patescibacteria. Strain PMX.108^T^ was isolated from mesophilic methanogenic sludge in an anaerobic laboratory-scale bioreactor treating synthetic purified terephthalate- and dimethyl terephthalate-manufacturing wastewater. The strain could not grow axenically and is obligately anaerobic and parasitic, strictly depending on *M. hungatei* as a host. The genome was comparatively large (1.54 Mbp) compared to other members of the clade, lacked some genes involved in the biosynthesis pathway and encoded type IV pili-related genes associated with the parasitic lifestyle of ultrasmall microbes. The G+C content of the genomic DNA was 36.6 mol%. Here, we report the phenotypic and genomic properties of strain PMX.108^T^; we propose *Minisyncoccus archaeiphilus* gen. nov., sp. nov. to accommodate this strain. The type strain of the species is PMX.108^T^ (=JCM 39522^T^). We also propose the associated family, order, class and phylum as *Minisyncoccaceae* fam. nov. *Minisyncoccales* nov., *Minisyncoccia* class. nov. and *Minisyncoccota* phyl. nov. within the bacterial kingdom *Bacillati*.

## Introduction

*Candidatus* Patescibacteria or the candidate phyla radiation (CPR) is a large bacterial phylogenetic group that includes various lineages of uncultivated bacteria [1,2]. While the phylogeny and taxonomy remain debated, there is a general consensus that these bacteria are characterized by small cell sizes (~0.2 µm on average diameter) [3], small genomes (<1 Mbp on average) [4,5] and incomplete biosynthesis pathways (e.g. nucleic acids, amino acids and lipids) [6,8]. These characteristics are associated with the organisms’ parasitic or predatory lifestyles [8,10]. Symbioses of *Ca*. Saccharimonadia/*Ca*. Saccharimonadota (formerly known as TM7 group) or *Ca*. Gracilibacteria/*Ca*. Altimarinota reported to date include intra-domain interactions with *Actinomycetota* or *Gammaproteobacteria*, respectively [7,11,16]. In our recent studies, we obtained cultures enriched in members of *Ca*. Paceibacteria/*Ca*. Paceibacterota [2,16] (formerly known as *Ca.* Parcubacteria/OD1 group [1,17]) of *Ca*. Patescibacteria and archaea and, through the microscopic observations and gene expression analysis, uncovered novel parasitic relationships between them – i.e. parasitism of *Ca*. Yanofskyibacterium parasiticum on *Methanothrix* spp. [10,18] and *Ca*. Minisyncoccus archaeophilus/*Ca*. Microsyncoccus archaeolyticus on *Methanospirillum* spp. [9,10], each, respectively, belonging to previously proposed candidate taxa *Ca*. Yanofskyibacteriaceae (formerly *Ca*. Yanofskybacteria/UBA5738) and *Ca*. Minisyncoccaceae (formerly 32–520/UBA5633). However, no two-strain co-cultures for the archaeal symbionts have been established. In addition, while there are reports of isolates from *Ca*. Patescibacteria providing novel insight into the lifestyle of this lineage [6], no cultures are publicly available via culture collections, hindering progress in unravelling the biology of this unique and diverse bacterial clade.

In this study, we aimed to isolate and provide a full description of our previously proposed *Candidatus* taxa of *Ca*. Patescibacteria. We successfully isolated *Ca*. Minisyncoccus archaeophilus in a co-culture through the exclusion of other organisms by filtration and subsequent inoculation into a pure culture of the methanogen strain closest related to the host in the original mixed culture, *Methanospirillum hungatei* strain DSM 864^T^ (JF-1^T^). Here, we report the physiological properties of a novel archaea-parasitizing isolate designated strain PMX.108^T^ (=JCM 39522^T^) and propose the name *Minisyncoccus archaeiphilus* gen. nov. sp. nov. to accommodate the strain, as well as the associated family, order, class and phylum names in accordance with rules of the Internal Code of Nomenclature of Prokaryotes (ICNP) [19,20].

## Methods

### Cultivation

The inorganic basal medium was prepared as described previously [18]. Cultivation experiments were performed at 37 °C using 50-ml serum vials containing 20 ml of the medium under an atmosphere containing N_2_/CO_2_/H_2_ (~57:14:28, v/v). The culture media contained 1 mM acetate, 0.01% yeast extract (w/v), 0.05% casamino acid (w/v), 0.15% tryptone (w/v) and *Methanospirillum hungatei* strain DSM 864^T^ (2 ml/20 ml, medium). This methanogenic archaeon was the most frequently detected in the anaerobic sludge-derived enrichment cultures containing a high proportion of *Ca*. Minisyncoccus archaeophilus [9,10]. *Methanospirillum hungatei* strain DSM 864^T^ was pre-cultivated at 37 °C for 2 weeks using 10 mM acetate, 0.01% yeast extract (w/v) and 20 ml H_2_ gas.

The sludge samples containing strain PMX.108^T^ were collected from an anaerobic laboratory-scale bioreactor treating purified terephthalate- and dimethyl terephthalate-manufacturing wastewater in Sapporo, Hokkaido, Japan (43° 01′ 11″ N 141° 25′ 06″ E) [9,10, 18, 21]. The samples were inoculated into culture media containing substrates, such as ribonucleosides and amino acids according to previous studies [9,10, 18] and 5 mM acetate, formate, methanol and phenol. After several continuous cultivations and routine 16S rRNA gene-based analyses, strain PMX.108^T^ was the most enriched strain in the culture system with ribonucleosides, amino acids and phenol. To establish a co-culture of strain PMX.108^T^ with the host*,* this enrichment culture was purified by filtration with a 0.45-µm Millex-HV syringe filter (PVDF, 33 mm, gamma sterilized, Merck, Germany) to remove other cells, transferred to a medium containing *Methanospirillum hungatei* strain DSM 864^T^. To further select against non-target organisms, we also added ampicillin, vancomycin and streptomycin. These were chosen based on tests of nine antibiotics on the original enrichment culture to determine which antibiotics have less influence on PMX.108^T^ and more on other organisms. The purity of the co-culture of strain PMX.108^T^ and *Methanospirillum hungatei* strain DSM 864^T^ was checked by microscopic observations and 16S rRNA gene sequence analysis and confirmed by long-read shotgun DNA sequencing, as described below. After the establishment of the two-strain co-culture, we removed the antibiotics from the culture medium to avoid a negative influence on the growth of strain PMX.108^T^.

### Monitoring of the growth of the co-culture system

Cells of strain PMX.108^T^ were physically separated from the host methanogen by filtration [22] using a 0.45-µm Millex-HV syringe filter (PVDF, 33 mm, gamma sterilized, Merck, Germany), and 2 ml of filtrate was transferred to another culture system.

We prepared seven parallel culture systems of *Methanospirillum hungatei* strain DSM 864^T^ (H1-H7), strain PMX.108^T^ (M1-M7) and a co-culture (HM1-HM7) to monitor the growth of strain PMX.108^T^ and *Methanospirillum hungatei* strain DSM 864^T^. For 9 days after incubation, culture systems H1-H3, M1-M3 and HM1-HM3 were sampled daily (2 ml culture liquid per day) for DNA extraction, and culture systems H4, M4 and HM4 were sampled daily (1 ml culture liquid per day) for fluorescence *in situ* hybridization (FISH). Culture systems H5-H7, M5-M7 and HM5-HM7 were sampled daily (200 µl head space) for analysis by gas chromatography (GC-2014, Shimadzu, Japan, and GC-3210, GL Sciences, Japan) with a thermal conductivity detector fitted with a SHINCARBON-ST 50/80 stainless steel column (4.0×3.0 mm) (ID) as described in a previous study [18].

### Evaluation of optimal growth pH and temperature

To evaluate the optimal pH range for growth, 15 parallel cultures (duplicates) were prepared (pH 6.0, 6.1, 6.3, 6.5, 6.7, 6.9, 7.0, 7.1, 7.3, 7.5, 8.0, 8.5, 9.0, 9.5 and 10.0). The pH of the medium was adjusted with HCl or NaOH at room temperature. The co-culture of strain PMX.108^T^ and *Methanospirillum hungatei* strain DSM 864^T^ was sub-cultured (2 ml culture liquid) into fresh culture medium containing 1 mM acetate, 0.01% yeast extract (w/v), 0.05% casamino acid (w/v) and 0.15% tryptone (w/v). Two millilitres of culture liquid were sampled on days 0, 2, 6 and 11 for DNA extraction. To evaluate the growth temperature range and optimum, nine parallel cultures (duplicates) were prepared at pH 7 (15, 20, 25, 30, 37, 40, 45, 50 and 55 °C). Two millilitres of the culture liquid for DNA extraction were sampled on days 0, 2, 6 and 11. The growth under each condition was determined by quantitative PCR (qPCR) as described below.

### Host specificity test with various methanogens

To evaluate the host specificity of strain PMX.108^T^, eight parallel culture systems (in duplicate) of *Methanospirillum hungatei* strain DSM 864^T^, *Methanothrix soehngenii* strain DSM 3671^T^ (GP6^T^), *Methanolinea mesophila* strain DSM 23604^T^ (TNR^T^), *Methanosarcina barkeri* strain DSM 800^T^ (MS^T^), *Methanoculleus bourgensis* strain DSM 3045^T^ (MS2^T^), *Methanocella paludicola* strain DSM 17711^T^ (SANAE^T^), *Methanobacterium formicicum* strain DSM 2639 (JF-1), *Methanobrevibacter arboriphilus* strain DSM 1125^T^ (DH1^T^) and the co-culture of strain PMX.108^T^ and each methanogen were prepared. These methanogens belong to different methanogen orders (*Methanomicrobiales*, *Methanosarcinales*, *Methanocellales* and *Methanobacteriales*) that are frequently found in anaerobic bioreactors and were thus selected as potential hosts for evaluating the host specificity of strain PMX.108^T^. Cells of strain PMX.108^T^ were physically separated from the host methanogen by filtration as mentioned above, and 2 ml of filtrate was transferred to the cultures of each methanogen. After cultivation, 2 ml of the culture liquid for DNA extraction was sampled on days 0, 2, 4, 8, 15 and 28. The growth under each condition was determined by qPCR as described below.

### Growth monitoring using qPCR for 16S rRNA genes

All culture liquids for DNA extraction were centrifuged at 17 750 ***g*** and stored at −80 °C. The qPCR template standards were constructed using Premix Taq Hot Start Version (Takara Bio Inc., Japan) according to the manufacturer’s protocol. The template standard for strain PMX.108^T^ was created from the co-culture by the primer set EUB338f [23]/1492 r_CPR (5′-TACCCGTGCCTTGTTACGACTT-3′, a slightly modified version of 1492 r [24]). Template standards for each methanogen were obtained from the pure cultures. The standards for *Methanospirillum hungatei* strain DSM 864^T^, *Methanothrix soehngenii* strain DSM 3671^T^, *Methanolinea mesophila* strain DSM 23604^T^, *Methanosarcina barkeri* strain DSM 800^T^, *Methanoculleus bourgensis* strain DSM 3045^T^, *Methanocella paludicola* strain DSM 17711^T^ and *Methanobacterium formicicum* strain DSM 2639 were constructed via PCR using the primer set Arch21f/Arch1492r [25], and the standard for *Methanobrevibacter arboriphilus* strain DSM 1125^T^ was constructed using the primer set Arch349f [26]/Arch1492r. The constructed template standards were purified using a QIAquick Gel Extraction Kit (QIAGEN, Germany). The concentrations of the template standards were determined using a Qubit™ dsDNA High Sensitivity Assay Kit (Invitrogen, USA) to calculate the 16S rRNA gene copy number. All qPCR assays were performed using a StepOne Real-Time PCR System (Thermo Fisher Scientific, USA) with a MightyAmp™ for Real-Time (TB Green Plus) (Takara Bio Inc., Japan). The reaction mixture was prepared according to the manufacturer’s instructions. The qPCR conditions were as follows: initial denaturation at 98 °C for 2 min, followed by 40 cycles of denaturation at 98 °C for 10 s, annealing at 52 °C (strain PMX.108^T^) or 50 °C (methanogens) for 15 s and extension at 68 °C for 30 s. The primer sets 32-520-1066f [9,10]/1492 r_CPR and Arch349f/Arch806r [26] targeting the 16S rRNA gene specific to strain PMX.108^T^ and methanogens, respectively, were used.

### Genome sequence analysis

DNA was extracted from the microbial cells using the Extrap Soil DNA Kit Plus Ver.2 (BioDynamics Laboratory Inc., Japan) according to the manufacturer’s protocol. For 16S rRNA gene sequence analysis, 16S rRNA genes were amplified using Univ515f–Univ909r, according to a previous study [21]. PCR products were purified using a QIAquick PCR Purification Kit (Qiagen, Valencia, CA, USA) according to the manufacturer’s protocol. DNA sequencing was performed using the MiSeq Reagent kit v3 and MiSeq system (Illumina, San Diego, CA, USA). Raw 16S rRNA gene sequences were analysed using QIIME 2 ver. 2021.4 [27] according to a previous study [18]. Taxonomic classification was performed using the classification sklearn with the silva database version 138 [28] and Greengenes2 version 2020.10 [29]. The complete genome sequence of strain PMX.108^T^ was determined by long-read shotgun sequencing using a PacBio Revio system (Pacific Biosciences, USA). Genomic DNA in the DNA extracts was purified using a QIAquick Gel Extraction Kit (QIAGEN, Germany). DNA libraries were constructed using the SMRTbell gDNA Amplification Kit and the SMRTbell Express Template Prep Kit 2.0 (Pacific Biosciences, USA). Raw sequence reads were analysed using the Aviary software package v0.8.3, with assembly (longread-type hifi), recovery and annotation options [30]. Quality checks and trimming were performed using fastp v0.23.4 with the default parameters [31]. The trimmed sequences were assembled using Flye 2.9.3-b1797 [32]. Assembled contigs were bound using Rosella ver.0.5.3 [33], MetaBAT and MetaBAT2 ver. 2.15 [34], MaxBin2 ver. 2.2.7 [35], SemiBin ver. 2.02 [36], Vamb ver. 3.02 [37] and CONCOCT ver. 1.10 [38]. To recover high-quality genomes, the automated program Das Tool ver. V2.6.3, which integrates a flexible number of binning results for bin optimization, was used [39]. Overlapping sequences of strain PMX.108^T^ were manually trimmed. Genomic quality and abundance analyses of the recovered genomes were performed using CheckM v1.2 [40], CheckM2 ver. 1.0.2 [41], CoverM ver. 0.7.0 [42] and SingleM v0.16.0 [43]. The taxonomic classification of the bins was conducted using GTDBtk v2.3.2 (GTDB release214; default parameters) [44]. The complete genome was automatically annotated using DFAST (https://dfast.ddbj.nig.ac.jp) [45,46], eggNOG-mapper (version emapper-v2.2.15–4-gb1fd458 based on eggNOG orthology data) [47,48], BlastKOALA [49], GhostKOALA [49], and DRAM software (--use_uniref option with default setting) [50].

### Phylogenetic analysis

Genome-based phylogenetic trees were constructed using concatenated 120 single-copy marker genes based on the marker sets of GTDB r214 [44] and replication/transcription/translation-related genes included in the bacterial marker proteins defined in GTDB r214 [51]. Conserved bacterial 120 marker genes of strain PMX.108^T^ were identified using ‘gtdbtk identify’ with default parameters and aligned to reference genomes using ‘gtdbtk align’ [44]. The replication/transcription/translation-related genes (PF00380.20, PF00410.20, PF00466.21, TIGR00019, TIGR00020, TIGR00029, TIGR00059, TIGR00061, TIGR00084, TIGR00116, TIGR00158, TIGR00166, TIGR00168, TIGR00362, TIGR00487, TIGR00580, TIGR00593, TIGR00635, TIGR00663, TIGR00922, TIGR01009, TIGR01011, TIGR01017, TIGR01021, TIGR01029, TIGR01032, TIGR01044, TIGR01059, TIGR01063, TIGR01066, TIGR01071, TIGR01079, TIGR01164, TIGR01169, TIGR01171, TIGR01391, TIGR01393, TIGR01632, TIGR01951, TIGR01953, TIGR02013, TIGR02027, TIGR02397, TIGR03625, TIGR03632, TIGR03654 and TIGR03953) were identified ‘gtdbtk identify’ with ‘--write_single_copy_genes’ option. The identified replication/transcription/translation-related marker gene sequences were aligned using MAFFT v7.475 (--localpair --maxiterate 1000) [52] and trimmed using BMGE v1.12 [53] with the BLOcks SUbstitution Matrix with less than 30% similarity (BLOSUM30), maximum gap rate of each position of 0.67 and minimum length of selected regions of 3 according to Nishihara *et al*., (2024) [51]. It has been reported that *Ca*. Patescibacteria is a member of the ‘Terrabacteria’ group and forms a sister clade with the phylum *Chlorofexota* [54,55]. Therefore, we analysed phylogenetic relationships between *Ca*. Patescibacteria, *Chloroflexota* and other bacterial phyla in the ‘Terrabacteria’ kingdom, *Bacillati*, which *Chloroflexota* belongs to [56]. Genomes for the tree reconstructions consisting of *Ca*. Patescibacteria, *Chloroflexota*, *Actinomycetota*, *Armatimonadota*, *Cyanobacteriota*, *Bacillota* and *Mycoplasmatota* were retrieved from the National Center for Biotechnology Information (NCBI) Genome database (https://www.ncbi.nlm.nih.gov/datasets/genome/) via the search filters of type strain/material and/or complete assembly level (Table S1, available in the online Supplementary Material). Genomes for the phylogenetic tree of *Ca*. Paceibacteria/Parcubacteria were retrieved using ‘gtdbtk align’ with taxonomic filters (--taxa_filter c__Paceibacteria) from the GTDB r214 [44]. Genome trees of *Ca*. Patescibacteria, *Chloroflexota*, *Actinomycetota*, *Armatimonadota*, *Cyanobacteriota*, *Bacillota*, *Mycoplasmatota* and *Ca*. Paceibacteria/Parcubacteria were constructed using IQ-TREE version 2.3.6 (-bb 1000) with one of the C10 to C60 profile mixture models (LG+CX0+G+F) estimated by ModelFinder [57]. For the 16S rRNA gene-based tree construction, we used nearly complete or full-length 16S rRNA gene sequences of genomes collected for *Ca*. Patescibacteria and the phyla *Chloroflexota*, *Actinomycetota*, *Armatimonadota*, *Cyanobacteriota*, *Bacillota* and *Mycoplasmatota*. The 16S rRNA gene sequences of the metagenome-assembled genomes were extracted using Prokka 1.14.6 [58]. Representative sequences were selected by clustering via CD-Hit v4.8.1 with a 90% cut-off [59]. Selected sequences were aligned using MAFFT v7.475 (--localpair --maxiterate 1000) [52]. A maximum likelihood tree was estimated using IQ-TREE version 2.3.6 (-bb 1000) with an optimal model chosen using ModelFinder (-m MFP). The genome and 16S rRNA gene sequences of phylum *Themotogota* were used as the outgroup for the phylogenetic tree reconstructions (see Table S1 for details). Transfer bootstrap support values of all phylogenetic trees were calculated by BOOSTER v0.1.2 [60]. Average nucleotide identities (ANIs) and average amino acid identities (AAIs) of complete or near-complete genomes were calculated using LZ-ANI v1.2.0 [61] and CompareM v0.1.2 (https://github.com/dparks1134/CompareM), respectively. Significant differences in the counted ANIs between/within selected phyla were calculated by Tukey’s Honest Significant Difference (HSD) test using R software version 4.4.0 [62].

### Fluorescence *in situ* hybridization

All culture liquids for FISH were centrifuged at 6000 ***g*** and fixed with 4% paraformaldehyde in PBS for 3 h at 4 °C. Fixed samples were stored in 50% ethanol with PBS at −20 °C. FISH was performed as previously described [63]. The fixed samples were hybridized with FISH probes on the glass slides at 46 °C for 3 h. After the hybridization step, the slides were washed at 48 °C for 20 min. Equimolar mixtures (EUB338mix) of EUB338 [64], EUB338I, EUB338II and EUB338III [65] were used to detect all bacteria. The formamide concentrations used in this study were as follows: EUB338mix, 10%; ARC915 [66] for all archaea, 35%; MG1200 [66] for the order *Methanomicrobiales*, 25%; and the previously designed 32-520-1066 probe for *Ca*. Minisyncoccaceae (formerly known as 32–520 lineages), including strain PMX.108^T^ [9,10], 30%. The 32-520-1066 probe covered 95.7%(22/23 match) of 32–520 lineages as a perfect match based on the silva138.1 database using TestProbe 3.0 [https://www.arb-silva.de/search/testprobe/] and perfectly matched the nearly full-length 16S rRNA gene sequences of strain PMX.108^T^ (LC715100) in the cultures, as described in a previous study [9,10]. All probes were labelled with FITC or Cy3. The FISH samples were stained with 4′,6-diamidino-2-phenylindole dihydrochloride (DAPI). The fluorescence of the FITC-labelled MG1200 probe disappeared when double staining of strain PMX.108^T^ and *Methanospirillum hungatei* strain DSM 864^T^ was performed, as described previously [9,10], We used 32-520-1066 and ARC915 probes for double staining of strain PMX.108^T^ and *Methanospirillum hungatei* strain DSM 864^T^, respectively. Microscopic images were obtained using an epifluorescence microscope (BX-53; Olympus, Tokyo, Japan) equipped with a colour CCD camera (DP-74; Olympus). Phase contrast, FISH and DAPI micrographic images were uniformly processed using Photoshop CC (Adobe) and ImageJ 1.53 k [67]. The FISH images were merged using the ImageJ software [67].

### Transmission electron microscopy

On cultivation day 3 of the co-culture, ~1 ml of culture medium was sampled and sandwiched with the copper discs in liquid propane at −175 °C. After the samples were frozen, the liquid was freeze-dried with 2% glutaraldehyde, 1% tannic acid in ethanol and 2% distilled water at −80 °C for 2 days. Dehydration was performed using anhydrous ethanol thrice for 30 min each. Infiltration was performed twice using propylene oxide for 30 min each, and the sample was placed in a 7:3 mixture of propylene oxide and resin for 1 h. After volatilization of propylene oxide, the sample was transferred to a fresh 100% resin and polymerized at 60 °C for 48 h. Ultrathin sections were cut to obtain a thickness of 70 nm with a diamond knife using an ultramicrotome (Ultracut UCT; Leica, Vienna, Austria). The sections were stained with 2% uranyl acetate at 25 °C for 15 min and washed with distilled water, followed by secondary staining with lead stain solution (Sigma-Aldrich Co., Tokyo, Japan) at room temperature for 3 min. The grids were observed with transmission electron microscopy (TEM) (JEM-1500Plus, JEOL Ltd., Tokyo, Japan) at 100 kV. Digital images were captured using a CCD camera (EM-14830RUBY2; JEOL Ltd., Tokyo, Japan).

### Scanning electron microscopy

On cultivation day 3 of the co-culture, ~1 ml of culture medium was sampled and pre-fixed with the mixed liquid containing 4% paraformaldehyde and 4% glutaraldehyde in 0.1M cacodylate buffer (pH 7.4) at 4 °C. Next, the sample was fixed with 2% glutaraldehyde in 0.1M cacodylate buffer (pH 7.4) at 4 °C overnight. Subsequently, the sample was fixed with 1% tannic acid in 0.1 M cacodylate buffer (pH 7.4) at 4 °C for 2 h. Finally, the sample was fixed with 2% osmium tetroxide in 0.1 M cacodylate buffer (pH 7.4) at 4 °C for 1 h. Dehydration was performed in the following series of steps: 50% ethanol at 4 °C for 30 min, 70% ethanol at 4 °C for 30 min, 90% ethanol under ambient temperatures for 30 min and anhydrous ethanol under ambient temperatures thrice for 30 min each. After these processes, dehydration was performed using anhydrous ethanol under ambient temperatures overnight. The dehydrated samples were placed in a 5:5 mixture of ethanol and t-butyl alcohol for 1 h at room temperature. The sample was then transferred to a t-butyl alcohol solution and treated thrice for 30 min each. Freeze drying was performed at 4 °C. The electroconductive coating was applied using an Osmium Plasma Coater (NL-OPC80NS; Nippon Laser and Electronics Laboratory) with a thickness of 30 nm. Images were captured using a scanning electron microscopy (SEM) (JSM-7500F; JFOL Ltd., Tokyo, Japan) at 3 kV.

### Deposition of DNA sequence data

The raw sequence data are available in the DRR Run (DRR609078) under the BioProject PRJDB18927. The complete genome of *Minisyncoccus archaeiphilus* strain PMX.108^T^ has been deposited in the DDBJ/GenBank/EMBL databases (AP038758). The full-length 16S rRNA gene sequence of *Minisyncoccus archaeiphilus* strain PMX.108^T^ is available in the DDBJ/EMBL/GenBank database (LC847185).

## Results and discussion

### Morphological characteristics of strain PMX.108^T^

**Fig. 1.**
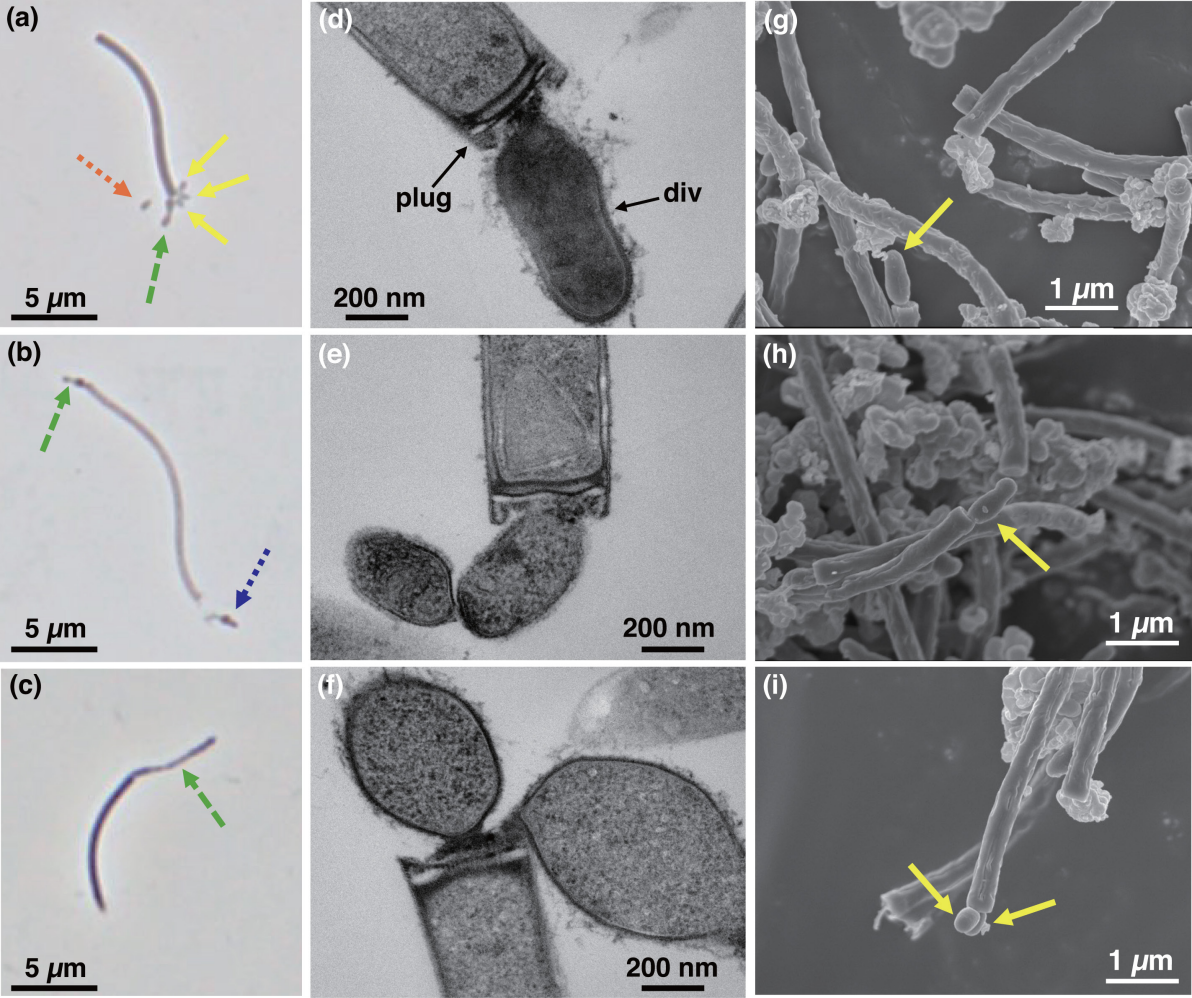
Micrographs of *Minisyncoccus archaeiphilus* strain PMX.108^T^ cells attached to *Methanospirillum hungatei* strain DSM 864^ T^ cells in the two-strain co-culture. (**a**)–(**c**) Phase-contrast microscopic images. (**d**)–(**f**) TEM images. (**g**)–(**i**) SEM images. The arrows in the images indicate strain PMX.108^T^ cells attached to strain DSM 864^ T^ cells (yellow solid), chained PMX.108^T^ cells (green dashed), detached PMX.108^T^ cells (blue dotted) and non-attached PMX.108^T^ cells (orange dotted). The plug and div on (**d**) indicate the plug structure of strain DSM 864^ T^ cells and dividing cells of strain PMX.108^T^, respectively.

**Fig. 2.**
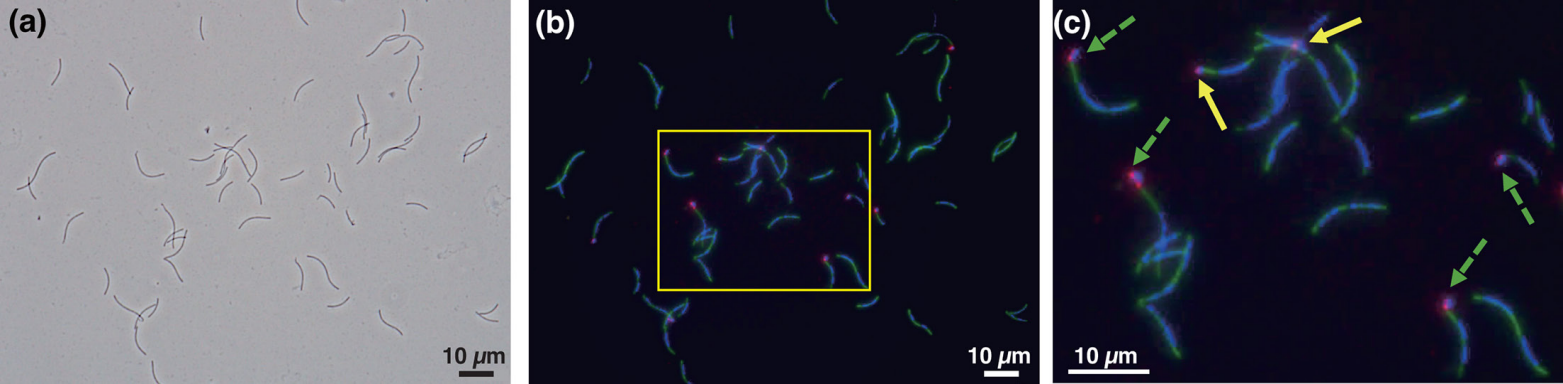
FISH observation of *Minisyncoccus archaeiphilus* strain PMX.108^T^ cells and *Methanospirillum hungatei* strain DSM 864^ T^ cells in the co-culture on day 4. (**a**) Phase-contrast micrographs. (**b**) and (**c**) Fluorescent microscopic images of cells stained with DAPI (blue), FITC-labelled ARC915 probe targeting strain DSM 864^T^ (green) and Cy3-labelled 32-520-1066 probe targeting *Candidatus* Minisyncoccaceae (magenta). (**c**) is an enlarged view of the boxed areas in (**b**). The arrows in the images indicate a single PMX.108^T^ cell attached to DSM 864^ T^ cells (yellow solid) and chained PMX.108^T^ cells (green dashed).

Strain PMX.108^T^ was isolated from anaerobic bioreactor sludge via enrichment in culture media supplemented with ribonucleosides, amino acids and phenol, filtration with a 0.45-µm filter to remove other cells, and inoculation into media containing active *Methanospirillum hungatei* strain DSM 864^T^ under anaerobic conditions at 37 ℃. Observation of cell morphology using phase-contrast microscopy, TEM and SEM showed that the strain PMX.108^T^ had a cell size of 0.73±0.10 µm in length and 0.46±0.06 µm in width and was frequently attached to the host’s plug structure as individual (Fig. 1a) or chains of cells (Fig. 1a, b) under the optimal cultivation conditions. The maximum length observed for the chains of cells was ~5 µm (Fig. 1c). In addition, non-attached PMX.108^T^ cells were frequently observed (Fig. 1b), suggesting that strain PMX.108^T^ has a ‘free-swimming’ stage to search for a new host. Such behaviour has also been observed for the ectosymbiotic *Ca*. Patescibacteria isolate TM7i [6] and the Diapherotrites, Parvarchaeota, Aenigmarchaeota, Nanoarchaeota and Nanohaloarchaeota (DPANN) archaeon isolate *Nanobdella aerobiophila* strain MJ1^T^ [68]. Given that isolate TM7i has been reported to use pili for host attachment and motility [6] and strain PMX.108^T^ possesses type IV pili-related genes (Table S2), pili may play an important role in the strain’s observed behaviour. Flagellar genes were not detected in the strain PMX.108^T^; therefore, the mechanisms supporting symbiosis/parasitism likely differ from those of *N. aerobiophila* strain MJ1^T^, which depends on archaella for host attachment and motility. Typically, within the first 2 days of co-cultivation of strain PMX.108^T^ and its host, individual cells could be observed attached to host cells. Subsequently, on the third and fourth days, host-attached cells were often found dividing or as chains of cells (Fig. 1b, e). Later in the incubation, detached PMX.108^T^ cells (usually as individuals and, in some cases, as chains) became observable (Fig. 1a, b). SEM showed that cells of strain PMX.108^T^ exclusively attached to the host’s plug structure (multilayered disc-shaped partitions) at the polar ends of the host filaments (Fig. 1g–i). No cells were observed attached to plug structures separating cells (i.e. in the middle of the filaments rather than the polar ends). Thus, attachment of PMX.108^T^ cells to its host archaeon may involve specific recognition of plug-associated surface structures. Based on TEM images, the host plugs with attached PMX.108^T^ cells often showed clear deformation with extracellular substances between the plug surface and attached PMX.108^T^ cells (Fig. 1d–f). Extracellular substances were also found surrounding the attached PMX.108^T^ cells. PMX.108^T^ cells that appeared to be dividing showed inward curving, which presumably corresponds to the division site (Fig. 1d). Observation of the cells using FISH and fluorescence microscopy revealed that PMX.108^T^ cells attached to the host archaeon showed much stronger fluorescence than those not attached (Fig. 2), suggesting that non-attached cells have lower ribosomal content than those attached to the host. Note that we cannot exclude the possibility that the cell walls of non-attached cells have lower permeability that limits penetration of the FISH probes.

### Growth characteristics of PMX.108^T^

**Fig. 3.**
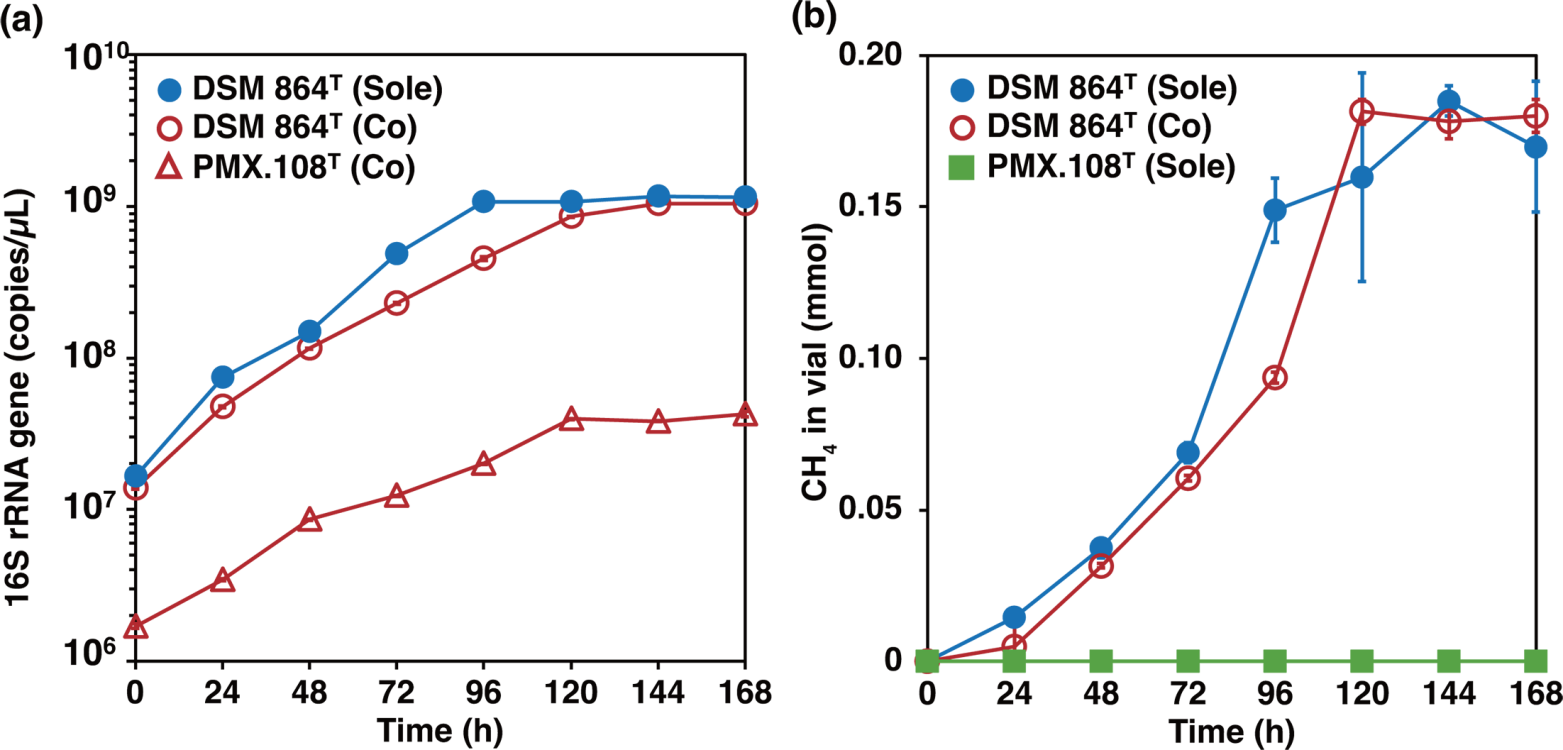
(**a**) Growth curves of *Methanorspirillum hungatei* strain DSM 864^T^ (DSM 864) and *Minisyncoccus archaeiphilus* strain PMX.108^T^. Pure cultures of strain DSM 864^T^ (blue circle with solid line) and co-culture of strains DSM 864^T^ (red frame circle) and PMX.108^T^ (red frame triangle) were monitored by qPCR for 16S rRNA genes (triplicate each). (**b**) Trends of methane production in the culture systems, pure cultures of DSM 864^T^ (blue circle with solid line), co-cultures of strains DSM 864^T^ and PMX.108^T^ (red frame circle) and strain PMX.108^T^ without its host strain DSM 864^T^ (green square with solid line) (triplicate each). ‘Sole’ and ‘Co’ in the figures indicate the sole inoculation of strain JF-1^T^ or strain PMX.108^T^ and the two-strain co-culture of strains DSM 864^T^ /PMX.108^T^, respectively. Error bars in (**a**) and (**b**) indicate sd.

PMX.108^T^ cells separated from its host archaeon via filtration neither showed growth nor methane production based on microscopic observation, qPCR targeting the 16S rRNA gene and monitoring of methane using gas chromatography. Co-incubation of filter-separated PMX.108^T^ cells with other methanogen species from seven other genera did not yield any growth (as measured by qPCR), indicating that, among the tested archaea, only *Methanospirillum hungatei* strain DSM 864^T^ can serve as the host (Table 1). Strain DSM 864^T^ co-incubated with strain PMX.108^T^ showed slower growth than in axenic culture but ultimately reached similar cell densities (Fig. 3a). Likewise, CH_4_ gas production by DSM 864^T^ was slower in the presence of strain PMX.108^T^ (Fig. 3b). Strain PMX.108^T^ grew at temperatures between 20 and 37 ℃ with optimum growth at 37 ℃. As for pH, growth occurred between 6.5 and 10.0 (optimum: 6.7–7.3) (Table 2). This coincides with the growth conditions of *Methanospirillum hungatei* strain DSM 864^T^ (Table 2) (optimum: 30–37℃ and pH 6.6–7.4) [69] and environmental conditions at which strain PMX.108^T^ was detected in the anaerobic bioreactor (pH 6.5–8.0 and temperature of 37℃) [21]. Note that the growth behaviour of strain PMX.108^T^ observed here may be highly influenced by the host organism’s growth behaviour.

**Table 1.** Host specificity of *Minisyncoccus archaeiphilus* strain PMX.108^T^ for methanogens

Taxonomy		Strain	Growth	
Family	Species		Methanogen	*Minisyncoccus archaeiphilus* strain PMX.108^T^
*Methanospirillaceae*	*Methanospirillum hungatei*	DSM 864^T^	+*	+
*Methanotrichaceae*	*Methanothrix soehngenii*	DSM 3671^T^	+	−
*Methanoregulaceae*	*Methanolinea mesophila*	DSM 23604^T^	+	−
*Methanosarcinaceae*	*Methanosarcina barkeri*	DSM 800^T^	+	−
*Methanomicrobiaceae*	*Methanoculleus bourgensis*	DSM 3045^T^	+	−
*Methanocellaceae*	*Methanocella paludicola*	DSM 17711^T^	+	−
*Methanobacteriaceae*	*Methanobacterium formicicum*	DSM 2639	+	−
*Methanobacteriaceae*	*Methanobrevibacter arboriphilus*	DSM 1125^T^	+	−

* The growth of *Methanospirillum hungatei* strain DSM 864T was inhibited in the co-culture with strain PMX.108T.

**Table 2.** Effects of growth temperature and pH on *Minisyncoccus archaeiphilus* strain PMX.108^T^

		Growth	
		*Methanospirillum hungatei* strainDSM 864^T^	*Minisyncoccus archaeiphilus* strain PMX.108^T^
Temperature (°C)	15	−	−
	20	+	+
	25	+	+
	30	+	+
	37	+	+
	40	−	−
	45	−	−
	50	−	−
	55	−	−
pH	6.0	+	−
	6.5	+	w*
	6.7	+	+
	6.9	+	+
	7.0	+	+
	7.1	+	+
	7.3	+	+
	7.5	+	w
	8.0	+	w
	8.5	+	w
	9.0	+	w
	9.5	+	w
	10.0	+	w

* Weak positive.

### Genomic characteristics

The total sequence length of the complete genome was 1 537 346 bp, with a DNA G+C content of 36.6 mol%. The genome encodes 1305 coding sequences (CDSs) (coding ratio: 91.5%), one copy each of the 5S, 16S and 23S rRNA genes and 44 tRNAs. As previously reported based on a draft genome of strain PMX.108^T^ [9,10], the genome lacks complete pathways for the tricarboxylic acid cycle, glycolysis, gluconeogenesis, pentose phosphate pathway and several genes relevant to biosynthetic pathways necessary for cell growth (e.g. biosynthesis of amino acids, nucleotides, cofactors, vitamins and fatty acids) (Table S2). The genome does not encode genes for a complete fatty acid biosynthesis pathway but harbours several genes encoding proteins that may facilitate several steps: acetyl-CoA carboxylase/biotin carboxylase subunit AccC (MNSC_05970), enoyl-[acyl-carrier protein] reductase II FabK (MNSC_05950), 3-oxoacyl-[acyl-carrier protein] reductase FabG (MNSC_05930), FabA-like domain protein (MNSC_05940) and long-chain acyl-CoA synthetase (MNSC_12360). How strain PMX.108^T^ synthesizes/acquires fatty acids and how these genes support that process remains unclear. Likewise, the strain PMX.108^T^ only encodes a partial pathway for the Embden–Meyerhof–Parnas pathway (interconversion of d-fructose 6-phosphate to glyceraldehyde 3-phosphate is lacking). As the genome encodes 2-oxoacid:ferredoxin oxidoreductase (MNSC_10430 and MNSC_10440), acetate---CoA ligase (MNSC_10230 and MNSC_10240), aldehyde dehydrogenase (MNSC_06270) and alcohol dehydrogenase (MNSC_08260) (Table S3), strain PMX.108^T^ may catabolize and ferment pyruvate to acetate and ethanol, respectively. The genome encodes genes for the F-type ATPase, which may facilitate the connection of cytosolic energy (ATP) and the membrane proton gradient [70].

### Phylogenetic analysis

**Fig. 4.**
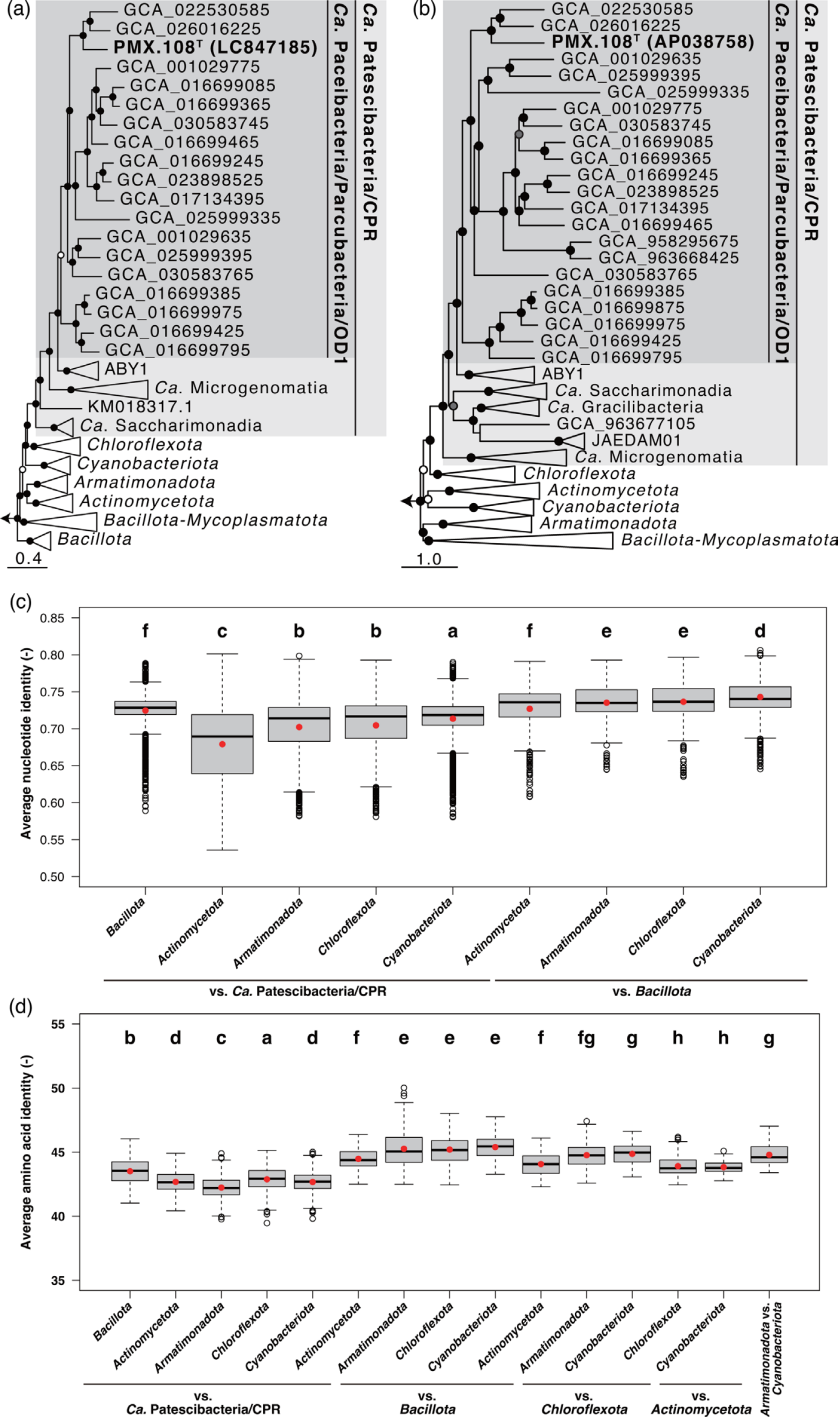
The maximum likelihood trees of *Minisyncoccus archaeiphilus* strain PMX.108^T^ based on (**a**) 16S rRNA gene sequences using IQ-TREE v. 2.3.6 with SYM+R7 model and (**b**) replication/transcription/translation-related proteins reconstructed using IQ-TREE v. 2.3.6 and box plots of the (**c**) ANIs and (**d**) AAIs between/within phyla. Branch supports are indicated with the following symbols: black circles for ≥95%, grey for ≥90% and white for ≥85% based on the 1000 ultrafast bootstrap replicates and BOOSTER-recalculated bootstrap values. The 16S rRNA genes and genomes of *Thermotogota* were used as the outgroups (not shown in the figure; see for details in Table S1 and Figs S2–S4). Different letters in (**c**) and (**d**) show significant differences based on Tukey’s HSD test (*P*<0.05), and white and red dots show outliers and ANI/AAI, respectively.

**Fig. 5.**
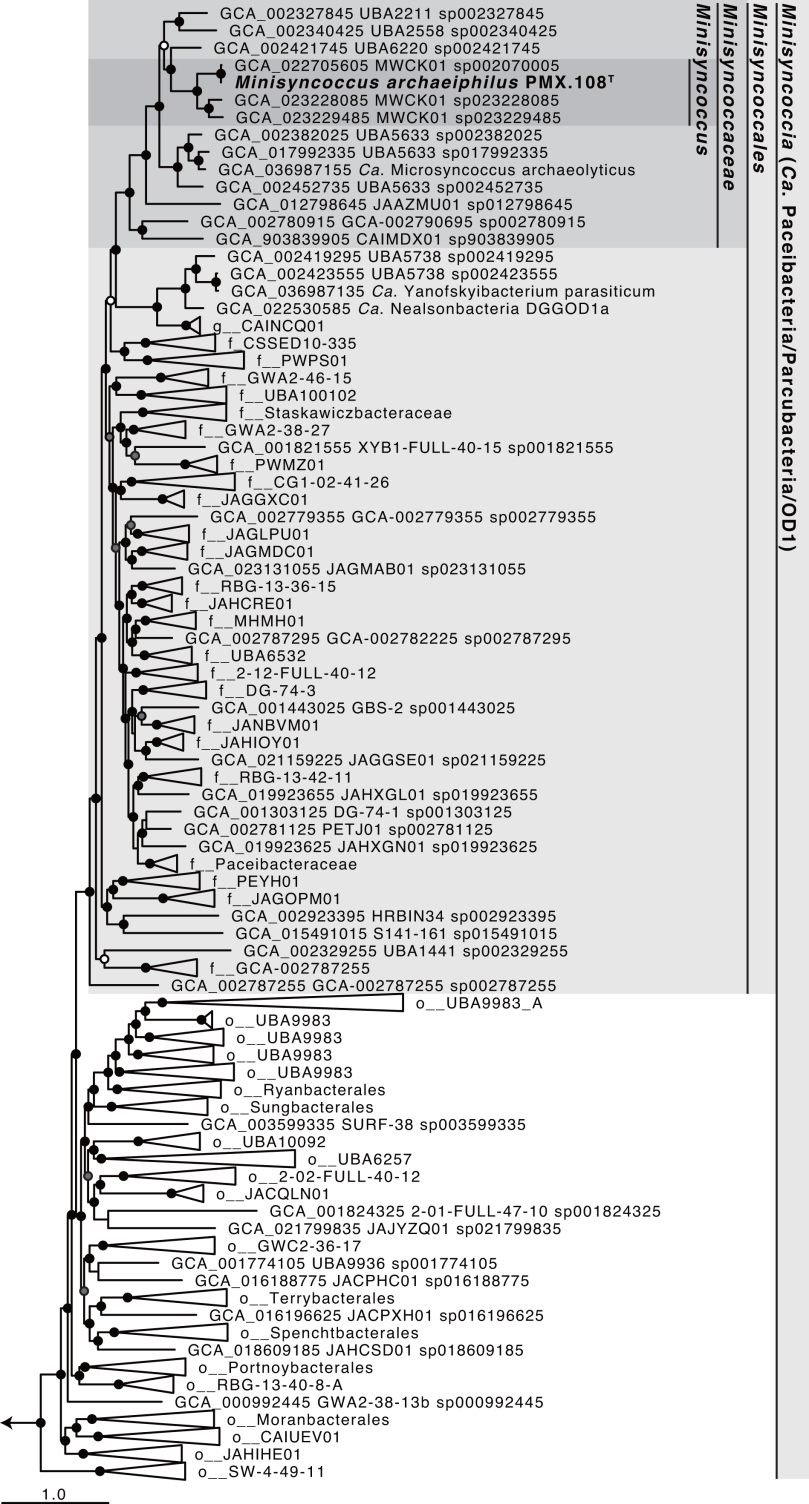
The maximum likelihood tree of *Minisyncoccus archaeiphilus* strain PMX.108^T^ based on conserved marker proteins using IQ-TREE v. 2.3.6. The concentrated alignment of 120 bacterial marker proteins provided by GTDB-tk (R214) was used for tree reconstruction. The order- (o__), family (f__), genus (g__)- and species-level classification according to GTDB r214 is shown for each genome. Branch supports are indicated with the following symbols: black circles for ≥95%, grey for ≥90% and white for ≥85% based on the 1000 ultrafast bootstrap replicates. The genomes of *Ca*. Saccharimonadia (GCA_000803625, GCA_037013405, GCA_030253535, GCA_030253515, GCA_005697565 and GCA000392435) were used as the outgroup (not shown in the figure).

The 16S rRNA gene of strain PMX.108^T^ has low sequence identity with those of type strains of validly described species, with the highest identity being 75.4% with the type strain of *Cryobacterium psychrophilum* strain DSM 4854^T^ of the phylum *Actinomycetota* and kingdom *Bacillati*. Note that, though isolates have been reported for *Ca*. Patescibacteria, none are publicly available via culture collections, and none have a validated species description. Amongst reported isolates of the lineage, the closest relative is *Ca*. Minimicrobia naudis strain IHU1 based on 16S rRNA gene identity (73.19%) [71]. Therefore, PMX.108^T^ belongs to a novel genus and species. In addition, strain PMX.108^T^ is the first cultured representative of a sublineage of *Ca*. Patescibacteria known as *Ca*. Paceibacteria/Parcubacteria [1,2, 17]. Phylogenetic analyses based on 16S rRNA gene sequences and bacterial marker proteins (120, as defined in the Genome Taxonomy Database (GTDB) [44], or a subset only including replication/transcription/translation-related proteins) all placed PMX.108^T^ as a member of the bacterial lineage corresponding to *Ca*. Patescibacteria and, more specifically, in a sublineage referred to as *Ca*. Paceibacteria or *Ca*. Parcubacteria (Figs 4a, b and S1–S4). In all cases, the clade including strain PMX.108^T^ branches within the kingdom *Bacillati* as a monophyletic group phylogenetically distinct from all other *Bacillati* phyla. More specifically, it branches as a sister group to the phylum *Chloroflexota*, a topology consistent with previous studies [54,55]. Collectively, this indicates that the lineage is at least a phylum-level lineage. In addition, given biological coherence in genotype – small genome size and the lack of many biosynthetic pathways – across the entire phylogenetic group and phenotype – dependency on parasitism and small cell size – across all cultured members, it is reasonable to conclude that the group forms a phylum. Moreover, these features clearly distinguish these organisms from other *Bacillati* phyla. ANIs between members of *Ca*. Patescibacteria and phylum *Bacillota* are not significantly different from ANIs between members of phyla *Bacillota* and *Actinomycetota* (*P*>0.05) (Fig. 4c). ANIs between members of *Ca*. Patescibacteria and the sistering phylum *Chloroflexota* are also not significantly different from the ANIs between *Ca*. Patescibacteria and a more distantly phylum *Armatimonadota* (*P*>0.05) (Fig. 4c). Though ANI is not a diagnostic metric for determining taxonomic rank, these are consistent with our inference that *Ca*. Patescibacteria forms a phylum-level taxonomic group. Comparison of AAIs also clearly distinguishes *Ca*. Patescibacteria from other *Bacillati* phyla (Fig. 4d). As reported in a previous study, taxonomic rank assignment based on relative evolutionary divergence in genome phylogeny also indicates that *Ca*. Patescibacteria consists of a single phylum [17]. While previous studies suggest that *Ca*. Patescibacteria contains multiple phyla [2,72, 73] based on a 16S rRNA gene sequence identity threshold of 75% [74], the highest 16S rRNA sequence identity observed between strain PMX.108^T^ and type strains in other phyla is very close to this threshold (75.36%), suggesting it may not be an appropriate metric and/or threshold in this case. Therefore, we propose that the bacterial lineage corresponding to *Ca*. Patescibacteria constitutes a phylum. This indicates that sublineages referred to as *Ca*. Paceibacteria/Parcubacteria, *Ca*. Saccharimonadia, *Ca*. Gracilibacteria, *Ca*. Microgenomatia and ABY1 are each equivalent to class-level lineages. This is consistent with the taxonomy defined in GTDB [17,44], which uses criteria and approaches distinct from this study for phylogenetic analyses. As such, we adopt GTDB classification as guidelines for defining the phylogenetic range of the lower taxonomic ranks (see Fig. 5) [9,10]. We also propose the associated family, order, class and phylum as *Minisyncoccaceae* fam. nov. *Minisyncoccales* ord. nov., *Minisyncoccia* class. nov. and *Minisyncoccota* phyl. nov with *Minisyncoccus* as the type genus.

## Note

Based on the Rule 30(3b) of ICNP, a type strain of the species must be deposited in at least two publicly accessible culture collections in different countries, with exceptional cases such as organisms requiring specialized facilities (e.g. Risk Group/Biological Safety Level 3 and high-pressure requirements) [19]. To date, *Pyrococcus yayanosii* strain CH1^T^ (JCM 16557^T^) [75] and *Promethearchaeum syntrophicum* strain MK-D1^T^ (JCM 39240^T^ and JAMSTEC no. 115508) [76] have been recognized as a species with validly published name due to the requirement of specialized equipment and difficulty of the detection by exceptionally low cell yield, respectively. Strain PMX.108^T^ also warrants consideration as an exception due to its fastidious nature, involving strong dependency on a host organism and the host’s condition. Owing to the efforts of the members of the Japan Collection of Microorganisms (JCM), Japan, we have successfully deposited the two-strain co-culture (JCM 39522^T^). Efforts are being made to complete the deposition of the strain PMX.108^T^ in a second recognized culture collection in another country.

Most validly published species names are based on axenic cultures, but Rule 31 a of the ICNP allows species description in regard to one member of unambiguous co-cultures (e.g. purified two-species cultures) [20]. Rule 31 a describes one example in which *Syntrophobacter wolinii* is one member of a syntrophic partnership with a hydrogen-consuming organism. Similar cases are documented for the following genera: *Pelotomaculum* [77,79], *Syntrophorhabdus* [80], *Microcaldus* [81], *Nanobdella* [82] and *Promethearchaeum* [76]. Thus, the description of *Microsyncoccus archaeiphilus* strain PMX.108^T^ for one member of the pure obligately symbiotic co-culture with *Methanospirillum hungatei* strain DSM 864^T^ is legitimate.

## Description of *Minisyncoccus* gen. nov.

*Minisyncoccus* (Mi.ni.syn.coc’cus. L. comp. masc. adj. *minor*, smaller, inferior; Gr. prep. *syn*, together; N.L. masc. n. *coccus*, coccus; from Gr. masc. n. *kokkos*, grain, seed; N.L. masc. n. *Minisyncoccus*, small coccus which lives together with another species). The genus belongs to the family *Minisyncoccaceae* of the order *Minisyncoccales*. The species type is *Minisyncoccus archaeiphilus*.

## Description of *Minisyncoccus archaeiphilus* sp. nov.

*Minisyncoccus archaeiphilus* (ar.chae.i’phi.lus. N.L. neut. pl. n. *Archaea*, a domain of prokaryotes; N.L. masc. adj. suff. -*philus*, friend, loving; N.L. masc. adj. *archaeiphilus*, *Archaea* loving). Cells are Gram-positive and exhibit cocci, irregular cocci or small rods. These structures are either present individually or attached to the plug structure of the host archaeon *Methanospirillum hungatei* strain DSM 864^T^. The cells are obligate parasites that grow under anaerobic conditions with their host. Growth occurs in the presence of acetate, yeast extract, casamino acids and tryptone in co-culture with the host. High concentrations of H_2_ gas may promote host growth, interfere with PMX.108^T^ parasitism and growth and destabilize the culture system. Under optimal growth conditions, chained or multiple cells are attached to a single plug structure of the host. The DNA G+C content was 36.6 mol% based on the genomic sequence. All strains sharing with the genome sequence of the type strain ANI values >95% are considered to be affiliated with this species. The type strain, PMX.108^T^ (=JCM 39522^T^), was obtained from *Candidatus* Patescibacteria-enriched culture in an anaerobic laboratory-scale bioreactor used to treat purified terephthalate and dimethyl terephthalate-manufacturing wastewater in Sapporo, Hokkaido, Japan. This species belongs to the genus *Minisyncoccus* and family *Minisyncoccaceae*. The complete genome and 16S rRNA gene sequences of *Minisyncoccus archaeiphilus* strain PMX.108^T^ were deposited in DDBJ/GenBank/EMBL under the accession numbers AP038758 and LC847185, respectively.

## Description of *Minisyncoccaceae* fam. nov.

*Minisyncoccaceae* (Mi.ni.syn.coc.ca.ce’ae. N.L. masc. n. *Minisyncoccus*, type genus of the family; *-aceae*, ending to denote a family; N.L. fem. pl. n. *Minisyncoccaceae*, the family of the genus *Minisyncoccus*). The family belongs to the order *Minisyncoccales* and class *Minisyncoccia*. The description of the family *Minisyncoccaceae* is the same as that of the genus *Minisyncoccus*. The type genus is *Minisyncoccus*.

## Description of *Minisyncoccales* ord. nov.

*Minisyncoccales* (Mi.ni.syn.coc.ca’les. N.L. masc. n. *Minisyncoccus*, type genus of the order; *-ales*, ending to denote an order; N.L. fem. pl. n. *Minisyncoccales*, the order of the genus *Minisyncoccus*). The order belongs to the class *Minisyncoccia* of the phylum *Mynisyncoccota*. The description of the order *Minisyncoccales* is the same as that of the genus *Minisyncoccus*. The type genus is *Minisyncoccus*.

## Description of *Minisyncoccia* class. nov.

*Minisyncoccia* (Mi.ni.syn.coc’ci.a. N.L. masc. n. *Minisyncoccus*, type genus of the class; -*ia*, ending to denote a class; N.L. neut. pl. n. *Minisyncoccia*, the class of the genus *Minisyncoccus*). The class belongs to the phylum *Minisyncoccota*. The description of the class *Minisyncoccia* is the same as that of the genus *Minisyncoccus*. The type genus is *Minisyncoccus*.

## Description of *Minisyncoccota* phyl. nov.

*Minisyncoccota* (Mi.ni.syn.coc.co’ta. N.L. masc. n. *Minisyncoccus*, type genus of the phylum; -*ota*, ending to denote a phylum; N.L. neut. pl. n. *Minisyncoccota*, the phylum of the genus *Minisyncoccus*). The phylum belongs to the kingdom *Bacillati*. The description of the phylum *Minisyncoccota* is the same as that of the genus *Minisyncoccus*. The type genus is *Minisyncoccus*.

## Supplementary material

10.1099/ijsem.0.006668 Uncited Supplementary Material 1.

10.1099/ijsem.0.006668 Uncited Supplementary Material 2.

## Abbreviations

AAIs: average amino acid identities
ANIs: average nucleotide identities
CDS: coding sequence
CPR: candidate phyla radiation
DAPI: 4′,6-diamidino-2-phenylindole dihydrochloride
FISH: fluorescence *in situ* hybridization
GTDB: genome taxonomy database
HSD: honest significant difference
ICNP: Internal Code of Nomenclature of Prokaryotes
JCM: Japan Collection of Microorganisms
NCBI: National Center for Biotechnology Information
qPCR: quantitative PCR
SEM: scanning electron microscopy
TEM: transmission electron microscopy
Tukey’s HSD: Tukey’s honest significant difference

## References

[1] Rinke C, Schwientek P, Sczyrba A, Ivanova NN, Anderson IJ (2013). Insights into the phylogeny and coding potential of microbial dark matter. Nature.

[2] Brown CT, Hug LA, Thomas BC, Sharon I, Castelle CJ (2015). Unusual biology across a group comprising more than 15% of domain bacteria. Nature.

[3] He C, Keren R, Whittaker ML, Farag IF, Doudna JA (2021). Genome-resolved metagenomics reveals site-specific diversity of episymbiotic CPR bacteria and DPANN archaea in groundwater ecosystems. Nat Microbiol.

[4] Tian R, Ning D, He Z, Zhang P, Spencer SJ (2020). Small and mighty: adaptation of superphylum Patescibacteria to groundwater environment drives their genome simplicity. Microbiome.

[5] Kagemasa S, Kuroda K, Nakai R, Li YY, Kubota K (2022). Diversity of *Candidatus* Patescibacteria in activated sludge revealed by a size-‍fractionation approach. Microbes Environ.

[6] Xie B, Wang J, Nie Y, Tian J, Wang Z (2022). Type IV pili trigger episymbiotic association of *Saccharibacteria* with its bacterial host. Proc Natl Acad Sci U S A.

[7] McLean JS, Bor B, Kerns KA, Liu Q, To TT (2020). Acquisition and adaptation of ultra-small parasitic reduced genome bacteria to mammalian hosts. Cell Rep.

[8] Castelle CJ, Brown CT, Anantharaman K, Probst AJ, Huang RH (2018). Biosynthetic capacity, metabolic variety and unusual biology in the CPR and DPANN radiations. Nat Rev Microbiol.

[9] Kuroda K, Kubota K, Kagemasa S, Nakai R, Hirakata Y (2022). Novel cross-domain symbiosis between *Candidatus* Patescibacteria and hydrogenotrophic methanogenic archaea *Methanospirillum* discovered in a methanogenic ecosystem. Microbes Environ.

[10] Kuroda K, Nakajima M, Nakai R, Hirakata Y, Kagemasa S (2024). Microscopic and metatranscriptomic analyses revealed unique cross-domain parasitism between phylum *Candidatus* Patescibacteria/Candidate Phyla Radiation and methanogenic archaea in anaerobic ecosystems. mBio.

[11] Fujii N, Kuroda K, Narihiro T, Aoi Y, Ozaki N (2024). Unique episymbiotic relationship between *Candidatus* Patescibacteria and *Zoogloea* in activated sludge flocs at a municipal wastewater treatment plant. Environ Microbiol Rep.

[12] He X, McLean JS, Edlund A, Yooseph S, Hall AP (2015). Cultivation of a human-associated TM7 phylotype reveals a reduced genome and epibiotic parasitic lifestyle. Proc Natl Acad Sci U S A.

[13] Batinovic S, Rose JJA, Ratcliffe J, Seviour RJ, Petrovski S (2021). Cocultivation of an ultrasmall environmental parasitic bacterium with lytic ability against bacteria associated with wastewater foams. Nat Microbiol.

[14] Moreira D, Zivanovic Y, López-Archilla AI, Iniesto M, López-García P (2021). Reductive evolution and unique predatory mode in the CPR bacterium *Vampirococcus lugosii*. Nat Commun.

[15] Yakimov MM, Merkel AY, Gaisin VA, Pilhofer M, Messina E (2022). Cultivation of a vampire: “*Candidatus* Absconditicoccus praedator”. Environ Microbiol.

[16] Oren A, Göker M (2023). *Candidatus* list. lists of names of prokaryotic *Candidatus* phyla. Int J Syst Evol Microbiol.

[17] Parks DH, Chuvochina M, Waite DW, Rinke C, Skarshewski A (2018). A standardized bacterial taxonomy based on genome phylogeny substantially revises the tree of life. Nat Biotechnol.

[18] Kuroda K, Yamamoto K, Nakai R, Hirakata Y, Kubota K (2022). Symbiosis between *Candidatus* Patescibacteria and archaea discovered in wastewater-treating bioreactors. mBio.

[19] Oren A (2023). Emendation of Principle 8, Rules 5b, 8, 15, 33a, and Appendix 7 of the International Code of Nomenclature of Prokaryotes to include the categories of kingdom and domain. Int J Syst Evol Microbiol.

[20] Oren A, Arahal DR, Göker M, Moore ERB, Rossello-Mora R (2023). International Code of Nomenclature of Prokaryotes. prokaryotic code (2022 revision). Int J Syst Evol Microbiol.

[21] Kuroda K, Narihiro T, Shinshima F, Yoshida M, Yamaguchi H (2022). High-rate cotreatment of purified terephthalate and dimethyl terephthalate manufacturing wastewater by a mesophilic upflow anaerobic sludge blanket reactor and the microbial ecology relevant to aromatic compound degradation. Water Res.

[22] Nakai R (2020). Size matters: ultra-small and filterable microorganisms in the environment. Microbes Environ.

[23] Hatamoto M, Imachi H, Ohashi A, Harada H (2007). Identification and cultivation of anaerobic, syntrophic long-chain fatty acid-degrading microbes from mesophilic and thermophilic methanogenic sludges. Appl Environ Microbiol.

[24] Turner S, Pryer KM, Miao VPW, Palmer JD (1999). Investigating deep phylogenetic relationships among cyanobacteria and plastids by small subunit rRNA sequence analysis. J Eukaryot Microbiol.

[25] DeLong EF (1992). Archaea in coastal marine environments. Proc Natl Acad Sci USA.

[26] Takai K, Horikoshi K (2000). Rapid detection and quantification of members of the archaeal community by quantitative PCR using fluorogenic probes. Appl Environ Microbiol.

[27] Bolyen E, Rideout JR, Dillon MR, Bokulich NA, Abnet CC (2019). Reproducible, interactive, scalable and extensible microbiome data science using QIIME 2. Nat Biotechnol.

[28] Yilmaz P, Parfrey LW, Yarza P, Gerken J, Pruesse E (2014). The SILVA and “all-species Living Tree Project (LTP)” taxonomic frameworks. Nucleic Acids Res.

[29] McDonald D, Jiang Y, Balaban M, Cantrell K, Zhu Q (2024). Greengenes2 unifies microbial data in a single reference tree. Nat Biotechnol.

[30] Newell RJP, Aroney STN, Zaugg J, Sternes P, Tyson GW (2024). Aviary: hybrid assembly and genome recovery from metagenomes with aviary.

[31] Chen S, Zhou Y, Chen Y, Gu J (2018). fastp: an ultra-fast all-in-one FASTQ preprocessor. Bioinformatics.

[32] Kolmogorov M, Yuan J, Lin Y, Pevzner PA (2019). Assembly of long, error-prone reads using repeat graphs. Nat Biotechnol.

[33] Newell RJP, Tyson GW, Woodcroft BJ Rosella: Metagenomic binning using UMAP and HDBSCAN.

[34] Kang DD, Froula J, Egan R, Wang Z (2015). MetaBAT, an efficient tool for accurately reconstructing single genomes from complex microbial communities. PeerJ.

[35] Wu YW, Simmons BA, Singer SW (2016). MaxBin 2.0: an automated binning algorithm to recover genomes from multiple metagenomic datasets. Bioinformatics.

[36] Pan S, Zhao XM, Coelho LP (2023). SemiBin2: self-supervised contrastive learning leads to better MAGs for short- and long-read sequencing. Bioinformatics.

[37] Nissen JN, Johansen J, Allesøe RL, Sønderby CK, Armenteros JJA (2021). Improved metagenome binning and assembly using deep variational autoencoders. Nat Biotechnol.

[38] Alneberg J, Bjarnason BS, de Bruijn I, Schirmer M, Quick J (2014). Binning metagenomic contigs by coverage and composition. Nat Methods.

[39] Sieber CMK, Probst AJ, Sharrar A, Thomas BC, Hess M (2018). Recovery of genomes from metagenomes via a dereplication, aggregation and scoring strategy. Nat Microbiol.

[40] Parks DH, Imelfort M, Skennerton CT, Hugenholtz P, Tyson GW (2015). CheckM: assessing the quality of microbial genomes recovered from isolates, single cells, and metagenomes. Genome Res.

[41] Chklovski A, Parks DH, Woodcroft BJ, Tyson GW (2023). CheckM2: a rapid, scalable and accurate tool for assessing microbial genome quality using machine learning. Nat Methods.

[42] Woodcroft BJ, Newell RJP, Aroney STN, Nissen J, Carmago A CoverM: read mapping statistics for metagenomics. https://github.com/wwood/CoverM.

[43] Woodcroft BJ, Aroney STN, Zhao R, Cunningham M, Mitchell JAM SingleM and sandpiper: robust microbial taxonomic profiles from metagenomic data. Microbiology.

[44] Chaumeil PA, Mussig AJ, Hugenholtz P, Parks DH (2020). GTDB-Tk: a toolkit to classify genomes with the genome taxonomy database. Bioinformatics.

[45] Tanizawa Y, Fujisawa T, Kaminuma E, Nakamura Y, Arita M (2016). DFAST and DAGA: web-based integrated genome annotation tools and resources. Biosci Microbiota Food Health.

[46] Tanizawa Y, Fujisawa T, Nakamura Y (2018). DFAST: a flexible prokaryotic genome annotation pipeline for faster genome publication. Bioinformatics.

[47] Cantalapiedra CP, Hernández-Plaza A, Letunic I, Bork P, Huerta-Cepas J (2021). eggNOG-mapper v2: functional annotation, orthology assignments, and domain prediction at the metagenomic scale. Mol Biol Evol.

[48] Huerta-Cepas J, Szklarczyk D, Heller D, Hernández-Plaza A, Forslund SK (2019). eggNOG 5.0: a hierarchical, functionally and phylogenetically annotated orthology resource based on 5090 organisms and 2502 viruses. Nucleic Acids Res.

[49] Kanehisa M, Sato Y, Morishima K (2016). BlastKOALA and GhostKOALA: KEGG tools for functional characterization of genome and metagenome sequences. J Mol Biol.

[50] Shaffer M, Borton MA, McGivern BB, Zayed AA, La Rosa SL (2020). DRAM for distilling microbial metabolism to automate the curation of microbiome function. Nucleic Acids Res.

[51] Nishihara A, Tsukatani Y, Azai C, Nobu MK (2024). Illuminating the coevolution of photosynthesis and Bacteria. Proc Natl Acad Sci U S A.

[52] Katoh K, Standley DM (2013). MAFFT multiple sequence alignment software version 7: improvements in performance and usability. Mol Biol Evol.

[53] Criscuolo A, Gribaldo S (2010). BMGE (Block Mapping and Gathering with Entropy): a new software for selection of phylogenetic informative regions from multiple sequence alignments. BMC Evol Biol.

[54] Coleman GA, Davín AA, Mahendrarajah TA, Szánthó LL, Spang A (2021). A rooted phylogeny resolves early bacterial evolution. Science.

[55] Beaud Benyahia B, Taib N, Beloin C, Gribaldo S (2025). Terrabacteria: redefining bacterial envelope diversity, biogenesis and evolution. Nat Rev Microbiol.

[56] Göker M, Oren A (2024). Valid publication of names of two domains and seven kingdoms of prokaryotes. Int J Syst Evol Microbiol.

[57] Minh BQ, Schmidt HA, Chernomor O, Schrempf D, Woodhams MD (2020). IQ-TREE 2: new models and efficient methods for phylogenetic inference in the genomic era. Mol Biol Evol.

[58] Seemann T (2014). Prokka: rapid prokaryotic genome annotation. Bioinformatics.

[59] Fu L, Niu B, Zhu Z, Wu S, Li W (2012). CD-HIT: accelerated for clustering the next-generation sequencing data. Bioinformatics.

[60] Lemoine F, Domelevo Entfellner J-B, Wilkinson E, Correia D, Dávila Felipe M (2018). Renewing Felsenstein’s phylogenetic bootstrap in the era of big data. Nature.

[61] Zielezinski A, Gudyś A, Barylski J, Siminski K, Rozwalak P (2024). Ultrafast and accurate sequence alignment and clustering of viral genomes. Bioinformatics.

[62] R Foundation for Statistical Computing VA R: A language and environment for statistical computing.

[63] Sekiguchi Y, Kamagata Y, Nakamura K, Ohashi A, Harada H (1999). Fluorescence *in situ* hybridization using 16S rRNA-targeted oligonucleotides reveals localization of methanogens and selected uncultured bacteria in mesophilic and thermophilic sludge granules. Appl Environ Microbiol.

[64] Amann RI, Binder BJ, Olson RJ, Chisholm SW, Devereux R (1990). Combination of 16S rRNA-targeted oligonucleotide probes with flow cytometry for analyzing mixed microbial populations. Appl Environ Microbiol.

[65] Daims H, Brühl A, Amann R, Schleifer K-H, Wagner M (1999). The domain-specific probe EUB338 is insufficient for the detection of all bacteria: development and evaluation of a more comprehensive probe set. System Appl Microbiol.

[66] Raskin L, Stromley JM, Rittmann BE, Stahl DA (1994). Group-specific 16S rRNA hybridization probes to describe natural communities of methanogens. Appl Environ Microbiol.

[67] Schneider CA, Rasband WS, Eliceiri KW (2012). NIH Image to ImageJ: 25 years of image analysis. Nat Methods.

[68] Gaisin VA, van Wolferen M, Albers S-V, Pilhofer M (2024). Distinct life cycle stages of an ectosymbiotic DPANN archaeon. ISME J.

[69] Ferry JG, Smith PH, Wolfe RS (1974). *Methanospirillum*, a new genus of methanogenic bacteria, and characterization of *Methanospirillum hungatii* sp.nov. Int J Syst Bacteriol.

[70] Chaudhari NM, Overholt WA, Figueroa-Gonzalez PA, Taubert M, Bornemann TLV (2021). The economical lifestyle of CPR bacteria in groundwater allows little preference for environmental drivers. Environ Microbiome.

[71] Ibrahim A, Maatouk M, Rajaonison A, Zgheib R, Haddad G (2021). Adapted protocol for *Saccharibacteria* cocultivation: two new members join the club of Candidate Phyla Radiation. Microbiol Spectr.

[72] Pallen MJ (2024). The dynamic history of prokaryotic phyla: discovery, diversity and division. Int J Syst Evol Microbiol.

[73] Anantharaman K, Brown CT, Hug LA, Sharon I, Castelle CJ (2016). Thousands of microbial genomes shed light on interconnected biogeochemical processes in an aquifer system. Nat Commun.

[74] Yarza P, Yilmaz P, Pruesse E, Glöckner FO, Ludwig W (2014). Uniting the classification of cultured and uncultured bacteria and archaea using 16S rRNA gene sequences. Nat Rev Microbiol.

[75] Birrien J-L, Zeng X, Jebbar M, Cambon-Bonavita M-A, Quérellou J (2011). *Pyrococcus yayanosii* sp. nov., an obligate piezophilic hyperthermophilic archaeon isolated from a deep-sea hydrothermal vent. Int J Syst Evol Microbiol.

[76] Imachi H, Nobu MK, Kato S, Takaki Y, Miyazaki M (2024). *Promethearchaeum syntrophicum* gen. nov., sp. nov., an anaerobic, obligately syntrophic archaeon, the first isolate of the lineage ‘Asgard’ archaea, and proposal of the new archaeal phylum *Promethearchaeota* phyl. nov. and kingdom *Promethearchaeati* regn. nov. Int J Syst Evol Microbiol.

[77] de Bok FAM, Harmsen HJM, Plugge CM, de Vries MC, Akkermans ADL (2005). The first true obligately syntrophic propionate-oxidizing bacterium, *Pelotomaculum schinkii* sp. nov., co-cultured with *Methanospirillum hungatei*, and emended description of the genus *Pelotomaculum*. Int J Syst Evol Microbiol.

[78] Qiu Y-L, Sekiguchi Y, Hanada S, Imachi H, Tseng I-C (2006). *Pelotomaculum terephthalicum* sp. nov. and *Pelotomaculum isophthalicum* sp. nov.: two anaerobic bacteria that degrade phthalate isomers in syntrophic association with hydrogenotrophic methanogens. Arch Microbiol.

[79] Imachi H, Sakai S, Ohashi A, Harada H, Hanada S (2007). *Pelotomaculum propionicicum* sp. nov., an anaerobic, mesophilic, obligately syntrophic, propionate-oxidizing bacterium. Int J Syst Evol Microbiol.

[80] Qiu Y-L, Hanada S, Ohashi A, Harada H, Kamagata Y (2008). *Syntrophorhabdus aromaticivorans* gen. nov., sp. nov., the first cultured anaerobe capable of degrading phenol to acetate in obligate syntrophic associations with a hydrogenotrophic methanogen. Appl Environ Microbiol.

[81] Sakai HD, Nur N, Kato S, Yuki M, Shimizu M (2022). Insight into the symbiotic lifestyle of DPANN archaea revealed by cultivation and genome analyses. Proc Natl Acad Sci U S A.

[82] Kato S, Ogasawara A, Itoh T, Sakai HD, Shimizu M (2022). *Nanobdella aerobiophila* gen. nov., sp. nov., a thermoacidophilic, obligate ectosymbiotic archaeon, and proposal of *Nanobdellaceae* fam. nov., *Nanobdellales* ord. nov. and *Nanobdellia* class. nov. Int J Syst Evol Microbiol.

