# Supplementary material for: Minisyncoccus archaeiphilus gen. nov., sp. nov., a mesophilic, obligate parasitic bacterium and proposal of Minisyncoccaceae fam. nov., Minisyncoccales ord. nov., Minisyncoccia class. nov. and Minisyncoccota phyl. nov. formerly referred to as Candidatus Patescibacteria or candidate phyla radiation
Source: Int J Syst Evol Microbiol. 2025 Feb 7;75(2):006668. doi: 10.1099/ijsem.0.006668 (PMC12281837; doi:10.1099/ijsem.0.006668)
Supplement: Uncited Supplementary Material 1. [file ijsem-75-06668-s001.pdf]

Minisyncoccus archaeiphilus gen. nov., sp. nov., a mesophilic, obligate parasitic bacterium, and proposal of *Minisyncoccaceae* fam. nov., *Minisyncoccales* ord. nov., *Minisyncoccia* class. nov., and *Minisyncoccota* phyl. nov. formerly referred to as *Candidatus* Patescibacteria or Candidate Phyla Radiation

– Supplementary figures–

Meri Nakajima[1,2], Ryosuke Nakai[1], Yuga Hirakata[3], Kengo Kubota[4,5], Hisashi Satoh[2], Masaru K. Nobu[6*], Takashi Narihiro[1,2*], Kyohei Kuroda[1,2*]

[1]Bioproduction Research Institute, National Institute of Advanced Industrial Science and Technology (AIST), 2-17-2-1 Tsukisamu-Higashi, Toyohira-ku, Sapporo, Hokkaido 062-8517, Japan
[2]Division of Environmental Engineering, Faculty of Engineering, Hokkaido University, North-13, West-8, Hokkaido 060-8628, Japan
[3]Bioproduction Research Institute, National Institute of Advanced Industrial Science and Technology (AIST), Central 6, Higashi 1-1-1, Tsukuba, Ibaraki 305-8566, Japan
[4]Department of Civil and Environmental Engineering, Graduate School of Engineering, Tohoku University, 6-6-06 Aza-Aoba, Aramaki, Aoba-ku, Sendai, Miyagi 980-8579, Japan
[5]Department of Frontier Sciences for Advanced Environment, Graduate School of Environmental Studies, Tohoku University, 6-6-06 Aza-Aoba, Aramaki, Aoba-ku, Sendai, Miyagi 980-8579, Japan
[6]Institute for Extra-cutting-edge Science and Technology Avant-garde Research (X-star), Japan Agency for Marine-Earth Science and Technology (JAMSTEC), 2-15 Natsushima-cho, Yokosuka, Kanagawa 237-0061, Japan

*Co-corresponding authors:
Kyohei Kuroda, Tel: +81 50 3522 8902; E-mail: k.kuroda@aist.go.jp
Takashi Narihiro, Tel: +81 50 3522 8889; E-mail: t.narihiro@aist.go.jp
Masaru K. Nobu, Tel: +81 46 867 9638; E-mail: mnobu@jamstec.go.jp

Supplementary Figures:

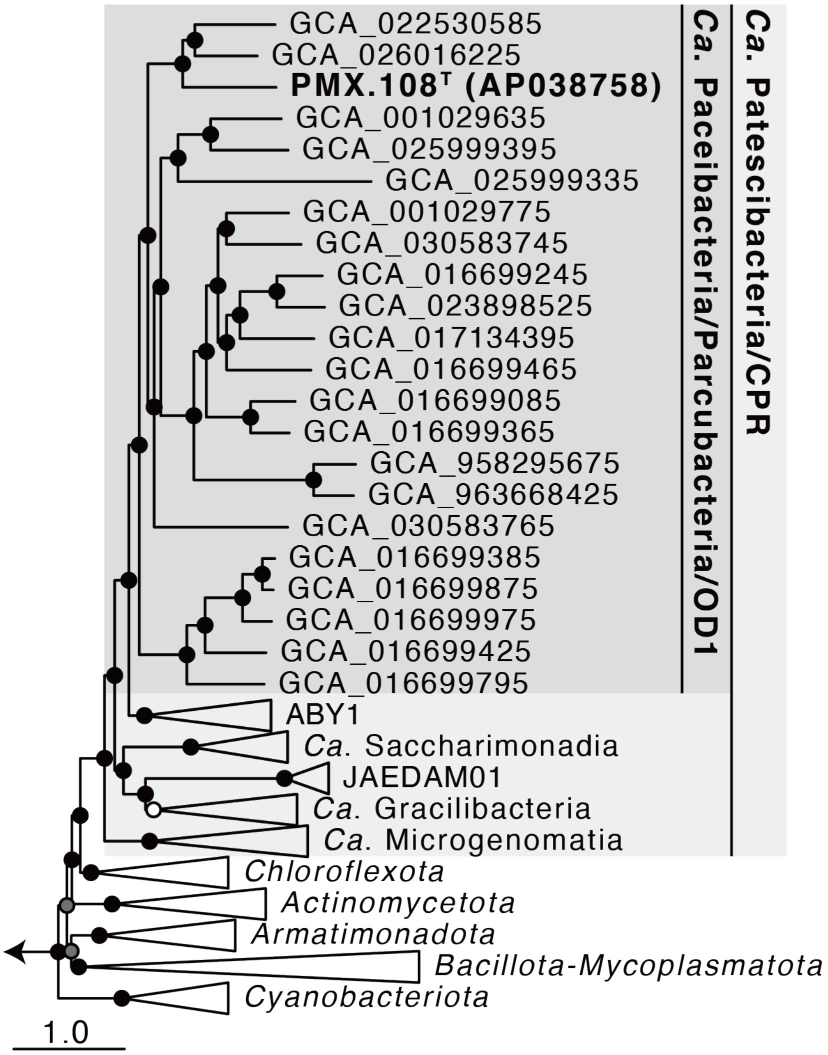

Figure S1 The maximum-likelihood tree of *Minisyncoccus archaeiphilus* strain PMX.108^T based on conserved bacterial 120 marker proteins based on GTDB-tk (R214) using IQTREE v. 2.3.6. Branch supports are indicated with the following symbols: black circles for ≥95%, grey for ≥90%, and white for ≥85% based on the 1,000 ultrafast bootstrap replicates and BOOSTER-recalculated bootstrap values. The genomes of *Thermotogota* were used as the outgroups (not shown in the figure, see for details in Table S1 and Fig. S4).

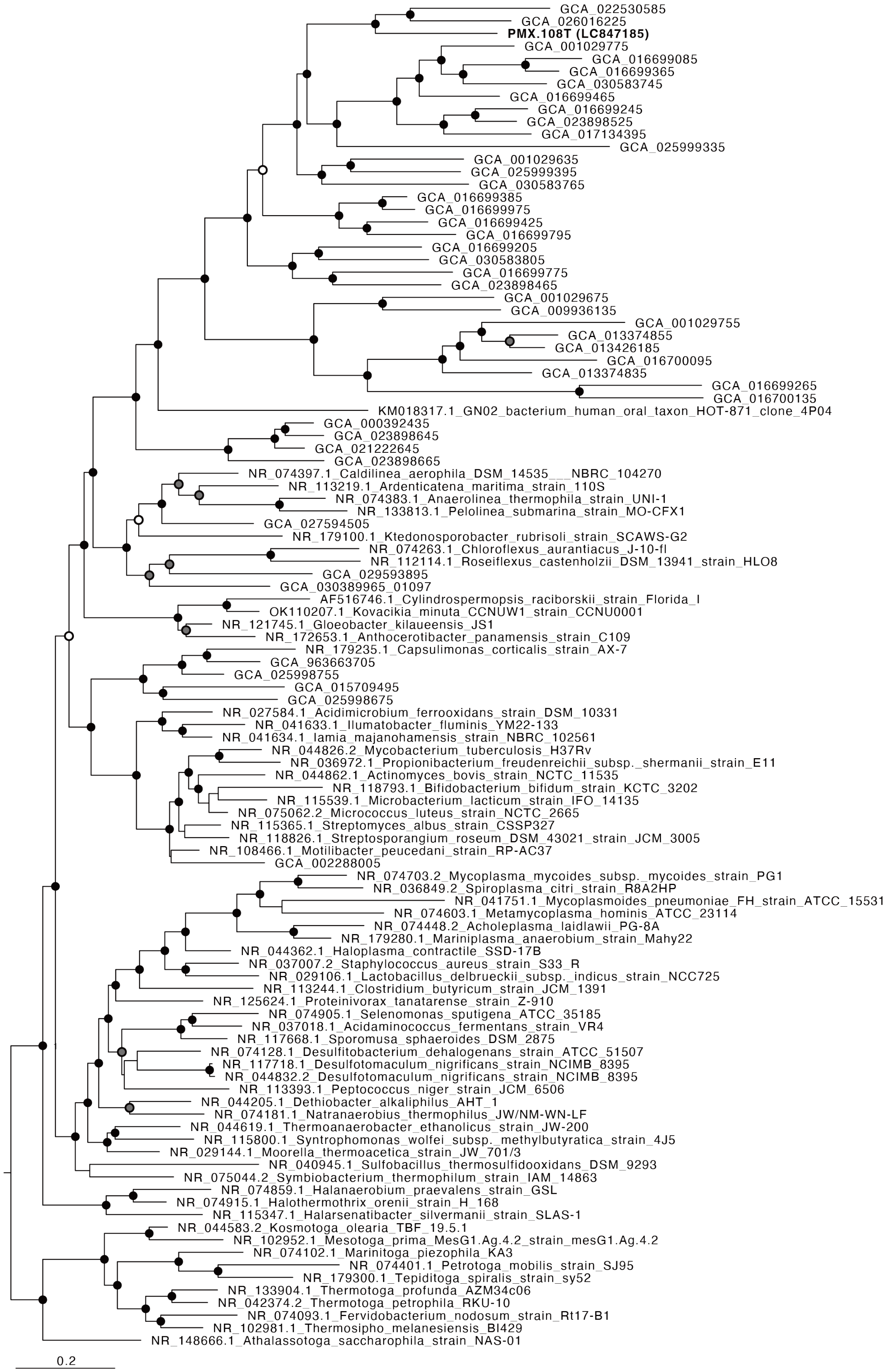

Figure S2 The maximum-likelihood tree of *Minisyncoccus archaeiphilus* strain PMX.108^{T} based on 16S rRNA gene sequences reconstructed using IQTREE v. 2.3.6 with SYM+R7 model (uncollapsed version of Figure 4A).

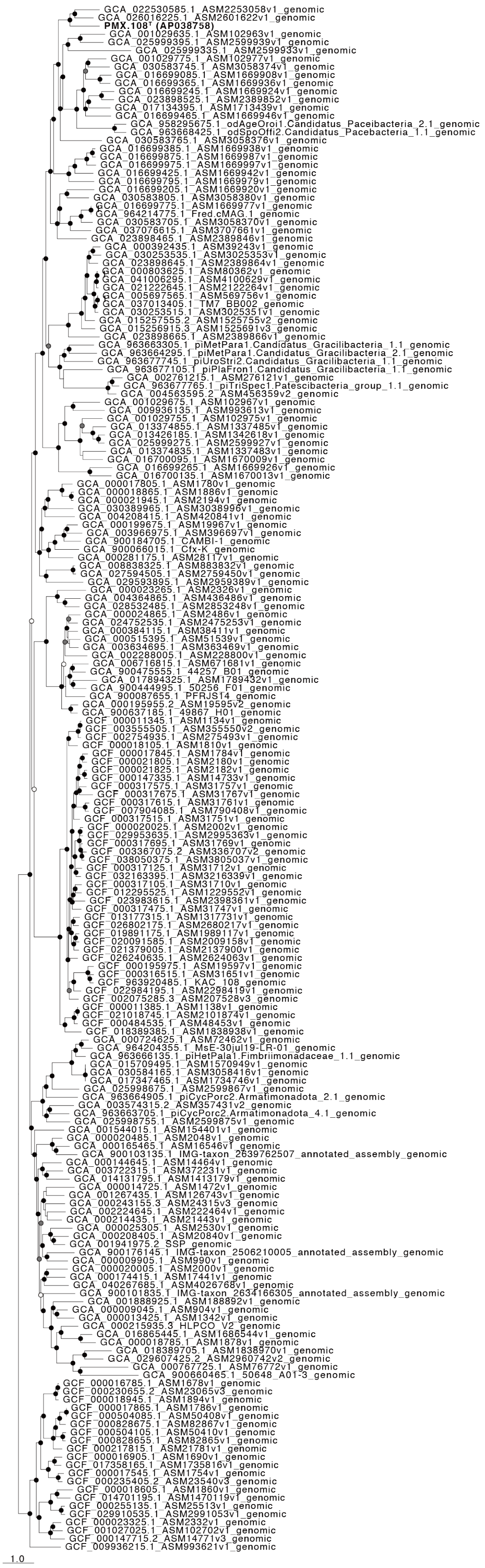

Figure S3 The maximum-likelihood tree of *Minisyncoccus archaeiphilus* strain PMX.108^T based on replication/transcription/translation-related proteins reconstructed using IQTREE v. 2.3.6 (uncollapsed version of Figure 4B).

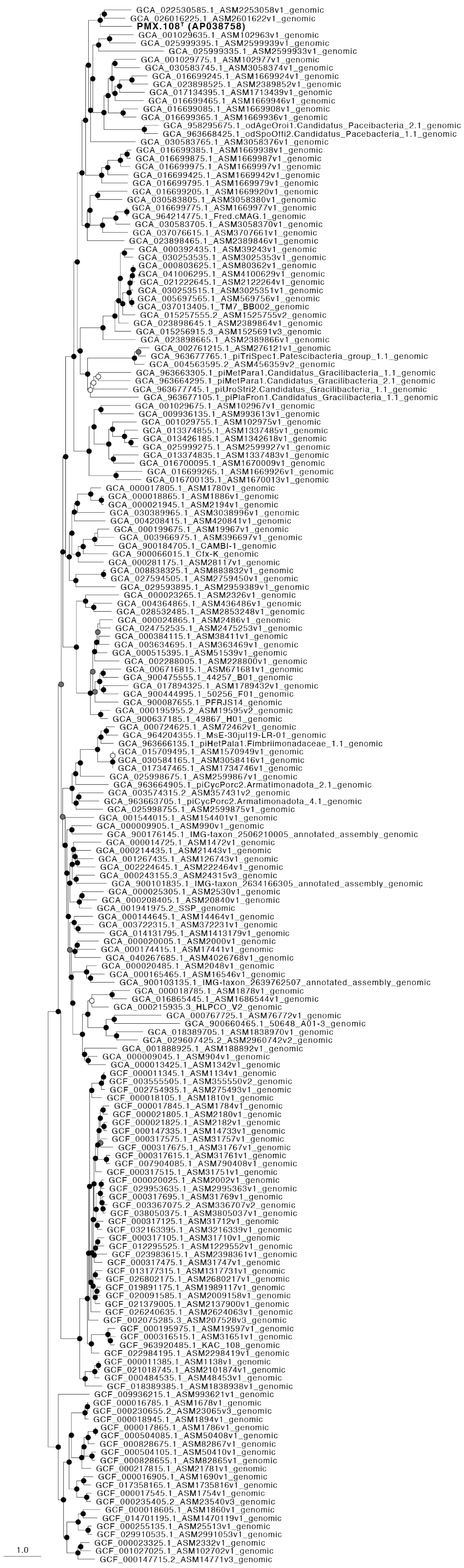

Figure S4 The maximum-likelihood tree of *Minisyncoccus archaeiphilus* strain PMX.108^{T} based on conserved bacterial 120 marker proteins based on GTDB-tk (R214) using IQTREE v. 2.3.6 (uncollapsed version of Figure S1).
